# The Potential Role of Exercise in Neuro-Oncology

**DOI:** 10.3389/fonc.2015.00085

**Published:** 2015-04-08

**Authors:** Prue Cormie, Anna K. Nowak, Suzanne K. Chambers, Daniel A. Galvão, Robert U. Newton

**Affiliations:** ^1^Edith Cowan University Health and Wellness Institute, Edith Cowan University, Joondalup, WA, Australia; ^2^School of Medicine and Pharmacology, University of Western Australia, Nedlands, WA, Australia; ^3^Department of Medical Oncology, Sir Charles Gairdner Hospital, Nedlands, WA, Australia; ^4^Griffith Health Institute, Griffith University, Southport, QLD, Australia; ^5^Cancer Council Queensland, Brisbane, QLD, Australia; ^6^Prostate Cancer Foundation of Australia, Sydney, NSW, Australia; ^7^The University of Hong Kong, Hong Kong, China

**Keywords:** exercise, physical activity, brain tumor, brain metastases, cancer

## Abstract

Patients with brain and other central nervous system cancers experience debilitating physical, cognitive, and emotional effects, which significantly compromise quality of life. Few efficacious pharmacological strategies or supportive care interventions exist to ameliorate these sequelae and patients report high levels of unmet needs in these areas. There is strong theoretical rationale to suggest exercise may be an effective intervention to aid in the management of neuro-oncological disorders. Clinical research has established the efficacy of appropriate exercise in counteracting physical impairments such as fatigue and functional decline, cognitive impairment, as well as psychological effects including depression and anxiety. While there is promise for exercise to enhance physical and psychosocial wellbeing of patients diagnosed with neurologic malignancies, these patients have unique needs and research is urgently required to explore optimal exercise prescription specific to these patients to maximize safety and efficacy. This perspective article is a discussion of potential rehabilitative effects of targeted exercise programs for patients with brain and other central nervous system cancers and highlights future research directions.

## Introduction

Malignant brain tumors and other central nervous system cancers (referred to as brain cancer hereafter) represent a highly challenging and devastating group of cancers. While the incidence of brain cancer is relatively low, mortality is high with the most recently reported 5-year survival rates at ~19–35% in Australia, United Kingdom, and America (1–3). Median survival varies with tumor pathology, grade, and demographic factors with older patients who have higher grade cancer and poor performance status faced with the worst prognosis of only limited months of survival (4). Not only does brain cancer have one of the lowest survival rates but it is also one of the leading sites contributing to burden of disease caused by cancer (1). Brain cancer patients experience debilitating physical, cognitive, and emotional effects, which significantly compromise quality of life (QOL) both for patients and their families (5, 6). The profound effect on physical and mental function leads to a premature loss of independence and significant economic burden both at the individual and societal level (7–9). This concern is accentuated by the fact that brain cancer is not just a disease of the elderly but occurs across all age groups, commonly affecting patients at the peak of their work and child-rearing responsibilities (average age of diagnosis is 57 years; ~65% of diagnoses occur at 45–84 years) (10). This paper focuses on the adult rather than pediatric setting.

At diagnosis, patients with brain cancer frequently experience neurological deficits including impaired balance, motor skills, and vision as well as headaches, seizures, and cognitive declines including memory and/or speech loss (11). In addition to tumor symptoms, treatment itself is associated with a range of toxicities. Treatment modalities include surgery, radiation, chemotherapy, and corticosteroids, alone and in combination (12). Some of the most troublesome adverse effects associated with these treatments include fatigue, myopathy, impairments in physical functioning, insomnia, additional cognitive decline, mood disturbance, and psychological distress (6, 13–20). Frequently used anticonvulsant medications may further accentuate fatigue and somnolence experienced by patients (21). As a consequence, patients are often unable to continue working and cannot legally drive. These issues combine to significantly compromise QOL (5). Currently, there are few established pharmacological strategies, which may ameliorate the debilitating effects associated with brain cancer and its treatment. Thus, not only do patients experience high symptom burden but also the symptoms are difficult to treat and based on the dearth of established management therapies may not be addressed. This is highlighted by the level of unmet supportive care needs among patients and their caregivers (22–24). Clearly, there is a pressing need to discover viable management options to counteract the incapacitating effects of brain cancer and address the unique needs of these patients. In this article, we discuss the potential of exercise as one such option.

## Exercise to Counteract Physical Effects of Brain Cancer

Brain cancer patients experience considerable physical impairments that compromise QOL and independence. The average level of fatigue experienced by these patients is ~40–50% more severe than normative levels for cancer patients, equating to approximately five times the clinically meaningful difference (25–29). Markedly reduced strength and fitness capabilities compared to age- and sex-matched norms have also been reported (26). Specifically, maximal muscular strength was observed to be 57 ± 28% of predicted values and cardiorespiratory fitness reported to be 41 ± 10% of predicted values among clinically stable patients following surgery (26). Functional capacity as assessed by the 6-min walk test has also been reported to be compromised in brain cancer patients, corresponding to 56 ± 13% of age- and sex-matched normative values (25). Notably, these data were collected in relatively well patients with good performance status (i.e., ≥70% Karnofsky performance status) and as such the degree of impairment for more debilitated patients is expected to be further compromised. Unfavorable changes in body composition are also apparent with a loss of lean mass and gains in fat mass evident following surgery (26). Additionally, while fatigue and somnolence are evident, insomnia is also commonly experienced by patients with brain cancer at higher rates than the general population (30).

To date, there have been no clinical trials evaluating the efficacy of exercise in counteracting the physical impairments experienced by brain cancer patients. However, clinical research has established the beneficial effect of exercise in ameliorating many of these impairments in other cancer populations. Specifically, appropriate exercise prescription has been shown to reduce cancer-related fatigue (31), enhance strength, fitness, and common functional movements (e.g., ambulation, chair rise, and stair climb ability) (32–35), promote favorable changes in body composition (i.e., increased lean mass and reduced fat mass) (33–35), and improve sleep quality (36). While these data were not specifically obtained from brain cancer patients, they provide strong theoretical rationale for the potential beneficial effect of exercise in these patients.

As of November 2014, there were no clinical trials registered in Australia, USA, or Europe, exploring the possible role of exercise in managing the side effects of brain cancer in adults. However, two small exercise intervention trials enrolling children with brain cancer have been recently launched in USA (NCT01944761, NCT02000986). Our team has been conducting pilot work to determine the feasibility and safety of exercise in adult grade III/IV glioma patients with initial observations suggesting that well-designed and appropriately supervised exercise may help to counteract many of the adverse physical side effects. To date, eight well-functioning (Eastern Cooperative Oncology Group performance status of 0–1) patients with high grade glioma have completed an exercise program involving three supervised exercise sessions weekly for the duration of chemoradiotherapy (~7 weeks) and are continuing with an additional 7 weeks of the exercise program after completing radiation therapy. Participants tolerated the exercise program well with no adverse events occurring during the exercise sessions and only one patient withdrawing due to time constraints. Despite intensive concurrent treatment, attendance was high at 87% and the average perceived exertion was in line with the target for people with cancer (13.4 ± 1.0 on the Borg 6–20 rating of perceived exertion scale; target = 12–16). Initial observations include 20 ± 16% improvement in muscle strength, 9 ± 10% improvement in cardiorespiratory fitness, and enhanced functional ability with improvements of 12 ± 7% in ambulation, 7 ± 11% in chair rise ability, and 12 ± 17% in dynamic balance. While only a short intervention period, favorable body composition changes were also observed with a 1.0 ± 2.4% average increase in lean muscle mass and varying results for changes in fat mass depending on whether or not patients were receiving dexamethasone (loss of 6 ± 6 vs. gain of 12 ± 12% fat mass in patients not receiving and receiving dexamethasone, respectively). Future research is needed to expand on this very early pilot work by exploring the efficacy of appropriately prescribed and supervised exercise for brain cancer patients in randomized controlled trials. Early results convey that dexamethasone use may be a relevant stratification factor and may require consideration in exercise prescription given the sequela of weight gain and proximal myopathy (37). It will also be important to investigate the potential role of exercise in patients with poorer performance status given the high demand for improving existing deficits and preventing further declines in physical functioning.

## Exercise to Counteract Cognitive Effects of Brain Cancer

Few patients avoid experiencing impairments in cognitive function associated with brain cancer (38–40). While the etiology and degree of impairment varies, declines commonly occur in memory, attention, executive function, verbal fluency, and visuospatial perception (39, 40). Such deficiencies significantly compromise QOL through adversely impacting daily activities and interpersonal relationships (41, 42). Impact on the carer and other family members’ QOL is considerable especially given the subsequent reductions in independence (39–42). While pharmacological and cognitive rehabilitation interventions have been proposed and show promising evidence, the management of cognitive effects caused by brain cancer and its treatment remains a major challenge (22, 43–45).

The potential role of exercise in attenuating such cognitive impairments has not been evaluated in brain cancer patients. However, a robust body of literature involving animal models as well as various patient models of healthy aging and other diseases associated with impaired cognition (e.g., Alzheimer’s disease, stroke) has established the efficacy of exercise as a potent therapy for maintaining and improving cognitive function (46–50). Exercise has a neuroprotective effect, reducing the risk of cognitive decline during aging as well as the incidence of dementia and Alzheimer’s disease (46–50). The vast majority of longitudinal research also indicates exercise is an effective intervention for improving cognitive function in cognitively healthy adults (46–50). Importantly, exercise has been observed to be particularly beneficial in reversing deficits among patients with cognitive impairments, resulting in improved cognitive function across a variety of domains (46–50). Findings from one of the first investigations specific to cancer suggest that exercise may also help counteract cognitive impairments caused by chemotherapy agents (based on an animal model of colorectal cancer) (51). A considerable body of literature including neuroimaging, human, and animal studies outside the cancer setting has elucidated the main mechanisms believed to be responsible for the preventative and restorative effects of exercise on cognition (52–55). Specifically, exercise mechanistically drives improvements in brain function and structure through stimulating neurogenesis and neural plasticity, up-regulating growth factors including brain-derived neurotropic factor, reducing levels of endogenous corticosteroids and pro-inflammatory cytokines, reducing oxidative stress, preserving brain volume, improving vascularization and increasing blood flow throughout the central nervous system, and increasing levels of hormones beneficial to neural structure and function (52–55). Despite clear differences in the pathophysiology of cognitive declines experienced by brain cancer patients, this establishes exercise as a promising intervention to counteract the cognitive effects experienced by patients.

Currently, there are a handful of registered trials evaluating the potential of exercise to prevent or rehabilitate cognitive impairments in cancer patients. The only investigation specifically involving people with brain cancer was recently opened in USA (NCT02153957) and will determine whether exercise improves cognitive problems in children treated with radiation at least 2 years prior to enrollment in the study. There are also two trials being conducted in adults with breast cancer; one led by our team exploring whether exercising during treatment prevents chemotherapy-induced cognitive impairment (ACTRN12614000051640) and the other ongoing in Canada investigating if exercise can improve cognitive dysfunction following the completion of chemotherapy (NCT01296893). While the results of these trials are pending, it is clear that specific research is required to evaluate the effects of exercise on cognitive function in patients with brain cancer. Based on evidence from other populations, future research should assess objective outcome measures of cognitive function including formal neurocognitive function testing, imaging studies, and self-report performance indicators from both patients and their carers. It would be beneficial to examine if exercise can delay onset and/or worsening of cognitive impairments as well as alleviate established deficiencies.

## Exercise to Counteract Emotional Effects of Brain Cancer

The diagnosis and treatment of brain cancer is undoubtedly a distressing experience, which has significant impact on psychosocial wellbeing (6, 20). There is a high prevalence of moderate to severe depression and anxiety among this patient group (20). In fact, the prevalence and severity of depression, anxiety, and overall emotional distress in people with brain cancer are consistently among the highest experienced for any cancer site (56). Beyond these well-defined disorders, patients experience a range of additional emotional challenges including existential issues, loss of self-identity, fear of and guilt about burden imposed on carers, stress, worry, uncertainty, loneliness, and a sense of waiting for cancer progression/death (57–60). The emotional effects of brain cancer extend to carers who also experience considerable declines in psychosocial wellbeing (61). Not surprisingly, the resulting negative impact on QOL is indeed significant (6, 62). Clearly, the psychosocial morbidity caused by brain cancer is profound and leads to a complex suite of supportive care needs for both patients and care-givers (63).

While there is a paucity of research investigating the potential of exercise in counteracting the emotional issues associated with brain cancer, there is clear evidence to suggest a potential therapeutic benefit. Most notably, exercise is recognized as a treatment option for the management of clinical depression by major psychological societies internationally (64, 65). These recommendations are informed by a series of meta-analyses establishing a significant positive effect of exercise in reducing depressive symptoms in adults without cancer (66–70). The efficacy of exercise in managing the psychological distress experienced by cancer patients has also been reported, with meta-analyses confirming the beneficial impact of exercise extends to people with cancer (71–73). A relatively small but statistically significant reduction in psychological symptoms has been observed although larger effects were reported for exercise programs that were supervised, clinic based, and involved a greater volume of exercise (71). Notably, the majority of participants were within the normal range on depression scales, raising the potential of more pronounced effects in distressed patients. The impact of exercise on other components of psychosocial wellbeing is rarely evaluated in existing literature but qualitative research provides evidence of considerable benefit across a range of psychosocial elements (74).

An interrelated group of biopsychosocial factors are theorized to be driving these exercise-induced improvements. Physiologically, exercise elicits favorable adaptations in endorphins, monoamine neurotransmitters (e.g., serotonin, dopamine and norepinephrine), neurotropic growth factors, inflammatory cytokines, and corticosteroids (47, 75–77). Additionally, specific exercise produces acute surges in testosterone in men and women, a powerful anabolic hormone with considerable non-genomic effects on the nervous system including reduced depression and anxiety (78–80). Furthermore, superior functional status is associated with lower depressive symptomology in brain cancer patients (81, 82), suggesting that exercise may also alleviate psychological distress by preserving physical capabilities and functional independence. There is a range of psychosocial factors that may also contribute including improved self-efficacy, social support provided by instructors, and peers involved with the exercise program and the potential of exercise to act as a distraction from negative thoughts (74, 83). Moreover, exercise is an intervention that patients have control of and it is possible that involvement in a structured exercise program may represent an opportunity to empower patients in dealing with the emotional impact of brain cancer (74).

Future research investigating the efficacy of exercise in counteracting the psychosocial morbidity associated with brain cancer is warranted for both patients and their carers. The potential complementary effect of exercise, psychological, and pharmacological interventions in counteracting these emotional problems poses an exciting avenue for novel research and superior clinical practice.

## Exercise and Survival

Higher performance status is a well-established prognostic factor in brain cancer, which is associated with better survival outcomes (4, 84). Given this relationship, it would seem intuitive that greater levels of exercise may also confer a protective effect against brain cancer progression and epidemiological evidence has suggested such an effect (85). Specifically, patients with brain cancer who achieved a greater volume of weekly aerobic exercise had a significantly reduced risk of mortality compared to those who exercise less (hazard ratio 0.64; 95% confidence intervals 0.46–0.91; *p* < 0.001) (85). This effect was independent of a range of prognostic factors including age, sex, grade, number of prior progressions, and performance status (85). The mechanisms driving this protective effect are unclear, but may involve a range of physiological adaptations that modulate tumor progression (86) and/or an enhanced ability to tolerate greater dosages of adjuvant treatment (87). However, it is possible that patients with superior exercise behavior have lower symptomology and as such these observations may reflect reverse causality rather than a physiological effect (85). While future research is required to elucidate the mechanisms, these data add to a growing body of literature reporting that exercise reduces the risk of cancer mortality in other patient groups (88–91). Additionally, it has been recently reported that increased aerobic exercise levels are associated with a reduced risk of fatal brain cancer (multivariate adjusted hazard ratio 0.58; 95% confidence intervals 0.35–0.95; *p* = 0.030), raising the hypothesis that exercise may also protect against the development of brain cancer (92).

## Implications for Practice

Exercise shows promise as a supportive care intervention to counteract the adverse impact of brain cancer. Despite their poor prognosis, a relatively high proportion of brain cancer patients participate in exercise throughout (59%) and after (69%) anticancer treatments (93). Furthermore, 60 and 76% of brain cancer patients are open to receiving information about exercise, and 66 and 91% of patients believe they may be able to participate in an exercise program during and after treatment respectively (94). In the absence of any brain cancer specific data, health professionals should rely on the current guidelines for all cancer patients when providing exercise advice to this group (95, 96). These guidelines recommend patients avoid inactivity even when undergoing difficult treatments and aim to participate in regular aerobic (e.g., walking, cycling) and resistance (i.e., lifting weights) exercise. To realize significant health benefits, patients should perform at least 150 min of moderate intensity aerobic exercise and two to three moderate intensity resistance exercise sessions weekly (95, 96). Decades of exercise science research have demonstrated that the quality of the exercise program, especially in terms of the mode, intensity, and volume of exercise, moderates the type and magnitude of adaptations in a dose-response fashion. Given this coupled with the complexity of physiological and psychological impairments common to people with brain cancer, sophisticated exercise prescription and monitoring are required. As such, referral to a clinical exercise physiologist is strongly recommended to ensure an appropriate exercise prescription that maximizes safety and patient benefits.

## Conclusion

There is strong theoretical rationale to suggest that exercise may be an effective intervention to aid in the management of brain cancer symptoms and treatment side effects. Clinical research has established the efficacy of appropriate exercise in counteracting physical impairments such as fatigue and functional decline, cognitive impairment, as well as psychological effects including depression and anxiety, within other cancer patients and chronic disease populations. This is supported by promising early evidence and clinical observations in brain cancer patients, but more research is required to explore optimal exercise prescription specific to the unique needs of these patients in order to maximize safety and efficacy. The potential rehabilitative effect of targeted exercise interventions in neuro-oncology is exciting, especially given that exercise represents a relatively inexpensive and highly accessible intervention that has very few adverse side-effects when appropriately prescribed and supervised.

## Conflict of Interest Statement

The authors declare that this research was conducted in the absence of any commercial or financial relationships that could be construed as a potential conflict of interest.
